# The Crucial Questions on Synovial Biopsy: When, Why, Who, What, Where, and How?

**DOI:** 10.3389/fmed.2021.705382

**Published:** 2021-08-06

**Authors:** Francesca Ingegnoli, Lavinia Agra Coletto, Isabella Scotti, Riccardo Compagnoni, Pietro Simone Randelli, Roberto Caporali

**Affiliations:** ^1^Division of Clinical Rheumatology, ASST Centro Specialistico Ortopedico Traumatologico Gaetano Pini-CTO, Milano, Italy; ^2^Department of Clinical Sciences & Community Health, Research Center for Adult and Pediatric Rheumatic Diseases, Research Center for Environmental Health, Università degli Studi di Milano, Milano, Italy; ^3^1° Clinica Ortopedica, ASST Centro Specialistico Ortopedico Traumatologico Gaetano Pini-CTO, Milano, Italy; ^4^Department of Biomedical, Surgical and Dental Sciences, Università degli Studi di Milano, Milano, Italy; ^5^Laboratory of Applied Biomechanics, Department of Biomedical Sciences for Health, Università degli Studi di Milano, Milano, Italy

**Keywords:** synovial biopsy, synovial membrane, rheumatoid arthritis, inflammatory arthritis, synovial analysis

## Abstract

In the majority of joint diseases, changes in the organization of the synovial architecture appear early. Synovial tissue analysis might provide useful information for the diagnosis, especially in atypical and rare joint disorders, and might have a value in case of undifferentiated inflammatory arthritis, by improving disease classification. After patient selection, it is crucial to address the dialogue between the clinician and the pathologist for adequately handling the sample, allowing identifying histological patterns depending on the clinical suspicion. Moreover, synovial tissue analysis gives insight into disease progression helping patient stratification, by working as an actionable and mechanistic biomarker. Finally, it contributes to an understanding of joint disease pathogenesis holding promise for identifying new synovial biomarkers and developing new therapeutic strategies. All of the indications mentioned above are not so far from being investigated in everyday clinical practice in tertiary referral hospitals, thanks to the great feasibility and safety of old and more recent techniques such as ultrasound-guided needle biopsy and needle arthroscopy. Thus, even in rheumatology clinical practice, pathobiology might be a key component in the management and treatment decision-making process. This review aims to examine some essential and crucial points regarding why, when, where, and how to perform a synovial biopsy in clinical practice and research settings and what information you might expect after a proper patient selection.

## Introduction

Changes in the organization of the synovial architecture are evident in the majority of joint diseases. Thus, the synovium has been studied at the macroscopic, microscopic, and molecular levels as it is an important determinant for the understanding of the biology of the joint and the etiopathogenesis of several joint diseases (1). In rheumatology, synovial tissue analysis provides insight into disease status and disease mechanisms by working as an actionable and mechanistic biomarker.

The synovium is a complex tissue composed of different cell types including tissue-resident macrophages, fibroblasts, and endothelial cells, as well as blood vessels, lymphatic vessels, and nerves (2). The histological analysis shows subcellular compartmentalization in two distinct zones: the lining layer and the sublining layer. The synovial lining has a crucial role in controlling the cellular and molecular exchange with the joint cavity and in maintaining joint integrity by regulating the composition of synovial fluid. In a healthy joint, it is made up of one to three cells thick and it is composed of tissue-resident macrophages and fibroblasts supported by a porous basement-like membrane, while the sublining, aside from fibroblasts and tissue-resident macrophages, includes nerves and blood and lymphatic vessels (2).

When pathology comes in, the normal architecture of the synovial membrane may be disrupted leading to alterations of the lining thickness, stromal cell density, and inflammatory infiltrate.

As in many joint diseases, the changes mentioned above occur early, and synovial tissue analysis might provide useful information for the diagnosis, especially in the case of atypical and rare joint disorders, and might have a supportive value in case of undifferentiated inflammatory arthritis, by improving disease classification. Moreover, it gives insight into disease progression helping patient stratification, a process in constant evolution. Finally, it contributes to an understanding of joint disease pathogenesis holding promise for the identification of new synovial biomarkers and the development of new therapeutic strategies (3).

This review aims to examine some essential and crucial points regarding why, when, where, and how to perform a synovial biopsy in clinical practice and research settings and what information you might expect after a proper patient selection. Given the breadth of the matter, we focus only on those aspects that are of the most interest to the rheumatologist.

## When Should the Synovial Biopsy Be Done?

**Figure 1:**
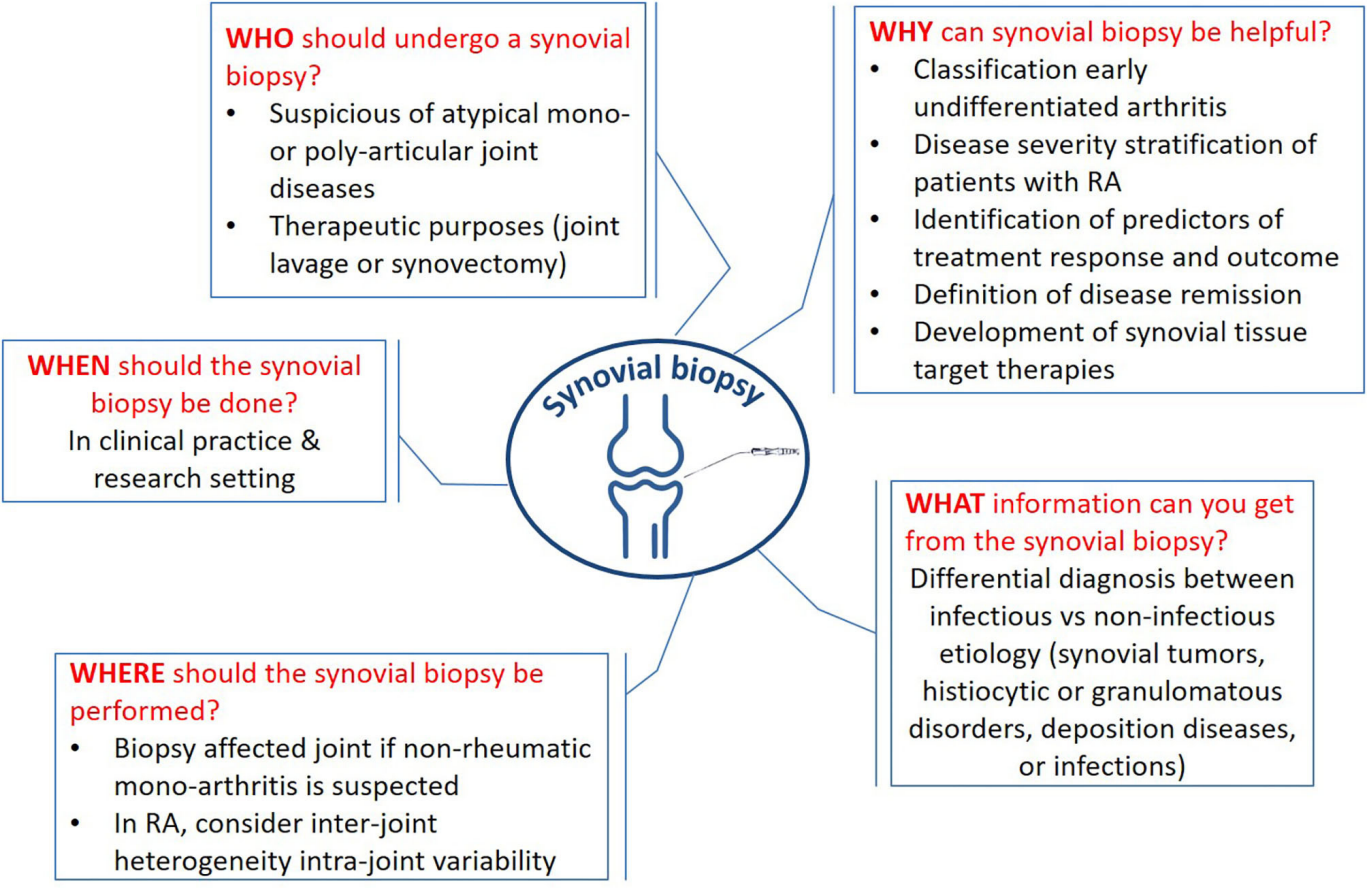
Key messages about the synovial biopsy. The figure sums up the crucial questions (why, when, what, who, where, and how) and the related answers.

The synovium is involved in all chronic inflammatory arthropathies. Although in routine clinical practice synovial biopsy is not mandatory for most diagnoses of inflammatory arthritis (e.g., rheumatoid arthritis—RA); in some circumstances it becomes irreplaceable (Figure 1). Indeed, when patient history, examination, and diagnostic investigations do not allow to delineate a clear picture and where there is a clinical suspicion of systemic forms, histological examination of synovial tissue with adequate sample processing can allow differential diagnosis between infective, neoplastic, deposition, and histiocytic diseases.

Moreover, a synovial biopsy can be complementary to the synovial fluid analysis. Comparative studies concerning the accuracy of the same diagnostic procedures (microbiological cultures, PCR for infective agents, crystals detection) on synovial fluid and synovial biopsy are not abundant but underline the utility of the two analysis, also in consideration of the possibility of false-negative results of both procedures (4–6).

A synovial biopsy is often performed for research purposes for example in RA patients; synovium histological and molecular alterations are considered a target for identifying new biomarkers to help rheumatologists tailor their clinical and therapeutic decision according to patient characteristics (7). Recently, a multicenter randomized control trial highlighted the possibility to integrate molecular pathology into clinical practice to improve treatment allocation of specific targeted therapies (8).

Finally, in the case of refractory synovitis (to local and systemic treatments), arthroscopic synovectomy could be a viable strategy to reduce local and persistent inflammation (9).

## Why Can Synovial Biopsy Be Helpful?

In rheumatology clinical practice, thanks to the great feasibility and safety of ultrasound-guided needle biopsy and needle arthroscopy, pathobiology may become a key component in the management and treatment decision-making process (10). For clinical and research purposes, histopathology and modern applications of molecular biology on synovial tissue are focused on the following major areas:

- ***Classification of early undifferentiated arthritis***. Since an early diagnosis and treatment of chronic inflammatory arthritis are linked to better long-term outcomes in terms of prevention of irreversible structural damage, nowadays the number of undifferentiated arthritis defined as inflammatory arthritis not satisfying classification criteria for RA (10, 11) is increasing. For this reason, an unmet need is the identification of biomarkers able to detect the patient who will develop RA or peripheral SpAs and differentiate them from those who will develop self-limiting or degenerative diseases. This would allow the use of the so-called “window of opportunity” for the more aggressive forms and, on the other hand, not to overtreat patients who will not develop chronic inflammatory arthritis. As far as concern the histological analysis, the cellular infiltrate and vascularity are informative. In a study of 95 patients with early (<1 year) unclassified arthritis, massive infiltration by CD38+ plasma cells and CD22+ B cells in the synovial sublining was able to predict the diagnosis of RA in the following 2 years of follow-up solely based on histological data with an accuracy of 85%. A diagnosis other than RA can be predicted in 97% of the cases when minimal infiltration by these cells was found (12). Previous research has identified as a possible distinctive marker to differentiate RA from spondyloarthropathies (SpA) and osteoarthritis (OA), the intensity of B and T cells infiltration (13). Several studies have found different characteristics in synovial vascularity among undifferentiated arthritis forms more prone to turn into RA or SpA. In the synovium of those who will develop SpA, blood vessels were increased in the sublining layer and more tortuous compared to the synovium of those who will develop RA (13–15). These findings are complementary to transcriptomic analysis. For example, r synovial markers suggested as specific for RA are the presence of intracellular citrullinated proteins and the differential expression of alpha-V integrin (13, 16). Moreover, angiogenic factors such as VEGF and Ang2 (mRNA and protein) were significantly more expressed in the synovial membrane of PsA than RA (17). Yeremenko et al. used pan-genomic microarrays of synovial samples and were able to recognize a myogene expression signature in SpA synovitis distinct from RA (18). Using a set of 100 transcripts on synovial tissue, based on their ability to discriminate RA from other inflammatory arthritic forms, Lauwerys et al. concluded that a diagnosis of RA can be predicted only by combining histological and clinical data (19). The study by Baeten et al. supports the validity of a multivariable prediction model by conjugating histological data (microscopic vascularity, lining layer thickness, assessment of synovial crystal deposition, staining for MHC-human cartilage gp39) with clinical and laboratory data to predict the evolution from undifferentiated arthritis into RA (20).

In particular, in the early disease stage, the presence of specific synovial histopathotypes defines distinct RA subtypes linked to diverse clinical phenotypes, disease activity/severity, and treatment response (21). These findings are further strengthened by the recent identification of different macrophage and fibroblast subsets in RA synovial tissue that are linked with different disease course and treatment responses (22).

- ***Disease severity stratification of patients with RA***. To date, the available risk stratification in RA patients is mainly represented by the presence of RF and ACPA autoantibodies together with CRP, the number of swollen joints at diagnosis, and the presence of erosive disease. Although valid, it certainly does not allow us to explain the great heterogeneity of the disease, prognosis, and response to treatments. Previous works have focused on RA prognosis in terms of disease severity/erosiveness. In a longitudinal study, the number of synovial lining layer macrophages at baseline correlates with the 1-year development of bone erosions in the hands and feet in patients with early (<18 months) inflammatory arthritis (mostly RA) (23). This finding was also confirmed in patients with established RA (24, 25). Furthermore, higher levels of MMP2 in synovial tissue samples from patients with early synovitis were correlated with the development of joint erosions (26). Previous studies have associated the presence of synovial lymphoid aggregates with the development of bone erosions (27), but subsequent studies on a larger number of patients did not confirm this result.
- ***Identification of predictors of treatment response and outcome***. Thanks to advances in molecular biology on synovial tissue, more recent studies have focused their attention on potential predictive synovial biomarkers of response to therapy. To date, unfortunately, we do not have tools to identify patients who are likely to benefit from a specific therapy. Dennis et al. identified four histological pathotypes confirmed by molecular analysis of gene-expression profile on synovial tissue in patients with RA: *lymphoid phenotype* characterized by enrichment of genes related to B cells and plasmablasts, and T lymphocyte activation and differentiation and antigen presentation; *myeloid phenotype* characterized by M1 monocyte signature with abundance of NFKB-dependent cytokines such as TNF-alpha and IL1-beta; *low-inflammatory phenotype*; and *fibroid phenotype* characterized by genes related to fibroblast and osteoclast/osteoblast regulation, and angiogenesis. In this study, the myeloid phenotype (associated with the circulating marker ICAM1) was more represented in the group of anti-TNF responder patients compared to the lymphoid pathotype (associated with the circulating marker CXCL13), which was more represented in IL6 inhibitor responders (28). In 2019, Humby et al. carried out histopathology and molecular analysis of synovial biopsies in a treatment-naive early RA patient cohort and demonstrated that the “myeloid” synovial pathological groups were most strongly correlated with a greater response to DMARD treatment as opposed to the “pauci-immune/fibroid” group, less responsive to treatment (29). In another treatment-naive early RA patient cohort, a baseline synovial “lymphoid-myeloid pathotype” was significantly associated with the requirement of bDMARD in the subsequent 12 months of follow-up (21). In a recently published study, the “pauci-immune phenotype” achieved a lower clinical response to certolizumab pegol in comparison with lymphoid-myeloid and diffuse-myeloid pathotypes (30). The results of Humby et al. showed that when anti-TNF inadequate responder patients with RA were classified as B cell-poor and B cell-rich by RNA sequencing on synovial biopsies, different responses to successive treatments were observed. While in patients defined as B cell-rich the efficacy of rituximab overlapped with tocilizumab, in the B cell-poor group tocilizumab was more efficacious than rituximab (8). However, studies did not always lead to univocal conclusions: it remains unclear if the response to treatment in RA is dictated by the presence of a marker of response to a specific agent or rather by the presence of a marker of disease severity, including disease duration and the number of previous DMARDs, and consequently a poor probability of response. In this regard, GADD45B expression (macrophage marker of disease severity) in synovial tissue in an early RA patient cohort was significantly higher in non-responders to methotrexate (MTX) or any first-line therapy (31).
- ***Definition of disease remission***. Finally, a synovial biopsy could represent an additional tool to define “real remission” in patients with RA or PsA. Despite apparent clinical remission, about 60% of patients have evidence of a residual power Doppler ultrasound (PDUS) positive synovitis at ultrasound evaluation (32– 34). This could explain the joint damage progression in these patients. For this reason, the concept of multidimensional remission has recently been introduced. It includes clinical parameters, PDUS or MRI, and normalization of synovium infiltrates. In particular, synovial mast cell density was independently associated with the clinical flare (35). Alivernini et al. showed that synovial histological features were comparable in patients with RA and PsA in clinical remission or low disease activity, despite being PDUS-negative. Residual synovitis persisted in PsA in clinical remission PDUS-negative patients (in terms of CD68+, CD3+, and CD31+). In this last scenario, treatment reduction or discontinuation would not appear safe in consideration of possible disease relapses (36). The analysis of possible prognostic biomarkers of disease relapse in patients with RA and PSA in remission is needed.
- ***Development of new targeted therapies***. Few studies focused on a possible synovial marker reflecting an early therapeutic effect in the target tissue after a short duration of therapy in RA. By studying serial biopsies (at least two for each patient), a significant result was achieved considering the reduction in the number of sublining CD68+ macrophages as a marker of the effectiveness of treatment independent of the primary mechanism of action (37, 38). This synovial marker could therefore allow an early-stage screening of new therapeutics development on a smaller number of subjects and accelerated decisions in phase I–II clinical trials. In this context, the use of standardized and validated techniques to detect and quantify CD68 macrophages and to obtain reliable results remains critical. Finally, thanks to investigation on synovial tissue biomarkers new targeted therapies have been identified as recently described in detail. These results have the role of improving a more innovative stratified trial design that improves the treatment decision-making (39).

## Who Should Undergo a Synovial Biopsy?

Patients requiring synovial biopsy represent a selected group in whom specific insights for the differential diagnosis workup of the joint disorder are needed to differentiate the numerous and various entities of atypical and rare mono- or poly-articular joint diseases, or those agreeable to biopsy for research purposes (Table 1). Synovium analysis is crucial in the diagnosis of monoarthritis and undifferentiated polyarthritis when synovial fluid cannot be aspirated. Moreover, when synovial tumors, histiocytic or granulomatous disorders, deposition diseases, or infections are suspected, synovial biopsy is often required. What can be seen in the biopsy specimen is directly dependent upon the sample processing and analysis performed, hence on the clinical suspicion selecting who is the patient deserving the procedure. Intending to address the dialogue between the clinician and the pathologist, below listed are few specific clinical findings peculiar to rare diseases with the corresponding histological pattern (Tables 2, 3).

**Table 1:** Differential diagnosis: WHO deserves synovial biopsy?

Infectious diseases	Presenting mainly with monoarthritis Common bacteria Mycobacterium tuberculosis Fungal arthritis Parasitic arthritis Lyme disease Presenting mainly with polyarthritis Whipple disease Mycobacterium leprae
Deposition diseases	Crystal arthropaties Ochronosis Hemochromatosis Amyloidosis
Systemic diseases	Sarcoidosis Multicentric reticulohistiocytosis
Synovial tumors	Synovial cell sarcoma/synovial chondrosarcoma Pigmented villonodular synovitis Synovial chondromatosis Lymphoma Metastatic carcinoma
Others	Foreign-body arthritis

**Table 2:** Main infectious etiologies for refractory monoarthritis: What do you find?

**Microorganism**	**Medium**	**Stains**	**Histology**
***Mycobacterium tuberculosis*** (40)	Agar-based and egg-based media incorporating green malachite and Middlebrook broths or solid media	Ziehl-Neelsen	Caseating or non-caseating granulomas
***Fungi*** (41)	Sabouraud's dextrose agar	Gomori methenamine silver, periodic acid Schiff	Candidiasis: thickened synovial membrane with non-specific mononuclear infiltration. Sporotricosis: mixed granulomatous and pyogenic processes. Rarely, asteroid bodies consisting of a central basophilic yeast surrounded by eosinophilic material radiating outward. Coccidioidosis: villonodular synovitis or typical pannus formation with non-caseating granulomas and sphreules containing coccidioidal endospores. Criptococcosis: both acute and chronic synovitis.
***Mycobacterium leprae*** (42)	Almost impossible to culture in a laboratory; PCR techniques for detecting DNA exist, but are currently not used in clinical practice.	Fite-Faraco staining	Non-specific granulomatous synovitis, epithelioid cells

**Table 3:** Main non-infectious etiologies for refractory mono- or poly-arthritis: WHAT do you find?

**Disease**	**Histology**
**Ochronosis** (43)	Paraffin-embedded sections show yellow-brown shards (able to provoke foreign body reactions with histiocytes and giants cells), scattered over the synovium and brittle pigmented articular cartilage. Haemosiderin and ochronotic pigment in macrophages, and focal inflammatory infiltrate of lymphocytes and plasma cells with some lining layer hyperplasia and hypervascularity may also be seen.
**Hemochromatosis** (44)	Low degree of synovial hyperplasia with mild infiltration of neutrophils, mononuclear cells -comprising macrophages- and lymphocytes; formation of synovial microvessels; haemosiderin deposition in the synovial lining cells; CPPD crystals may be seen.
**Amyloidosis** (45)	Diagnosis of amyloidosis requires Congo red staining to show amyloid deposits in the synovium. The immunohistochemical study allows typing of amyloidosis: antibodies directed against light chains of immunoglobulins (AL-amyloidosis), antibodies against the other major amyloid proteins (AA and ATTR).
**Multicentric reticulohistiocytosis** (46, 47)	Lipid-laden multinucleated giant cells and histiocytes with ground glass PAS-positive cytoplasm, which contains membrane-bound lysosomal granules, with a single large stellate Golgi apparatus.
**Sarcoidosis** (48, 49)	Histopathological examination of synovium reveals various patterns: diffuse infiltration with histiocytes and lymphocytes, mild lining cell proliferation, seldom vascular congestion. A granulomatous reaction is often absent.
**Crystal arthropathies** (50)	Gout and pseudogout: deposits of monosodium urate crystals or calcium pyrophosphate dihydrate crystals in the synovium after fixation with absolute alcohol and analysis with a polarization microscope, which shows to evaluate birefringence properties (negative MSU, positive CPPD), or DeGolanthal staining method. Basic calcium phosphate induced arthritis: crystals can be seen using alizarin red staining or transmission or scanning electron microscopy.

Finally, in some cases, arthroscopy might have also therapeutic purposes; for example, during the surgical procedure, a joint lavage might be useful to treat septic arthritis to remove crystal deposits and sometimes to benefit patients with active RA/PsA (51).

## What Information Can You Get From the Synovial Biopsy?

In the context of refractory monoarticular arthritis, among the infectious etiologies (Table 2), typical bacterial agents are more easily suspected, and in the event of unsuccessful isolation of the microorganism, broad-spectrum antibiotics are available. On the other hand, atypical microorganisms deserve special attention due to their less evocative clinical presentation, belated diagnosis, and the need for specific treatments based on the identification of the agent.

To start with, mycobacterium tuberculosis is a typical example of indolent and sometimes destructive arthritis (seldom of a prosthetic joint), where synovial biopsy and culture are required for the diagnosis and for selecting the right treatment regimen given the spread existence of multidrug-resistance bacteria. A detailed history should consider the following: previous TB exposure, living/traveling in endemic areas, concomitant HIV infection, previous trauma causing direct tissue inoculation, and concomitant TNF inhibitor therapy. These forms of arthritis follow a chronic course preferentially involving large joints (hip, knee) and rarely associate with constitutional symptoms or pulmonary findings (41, 52). Fungal arthritis caused by a hematogenous or contiguous spread in the setting of candidiasis, coccidiosis, blastomycosis, scedosporiosis, cryptococcosis, and sporotrichosis are not easily recognized. The patient is usually immunocompromised or of extremes ages, with a background of farm working, traveling in endemic zones, previous surgery, and comorbidities such as diabetes, alcoholism, or intravenous drug abuse. Arthritis again often involves the knee, ankle, elbow, or wrist, and clinical hints are the evidence of coexisting osteomyelitis and extra-articular manifestations of pulmonary and cutaneous relevance. In the case of sporotrichosis, tenosynovitis and bursitis may be present (53).

Parasites (giardiasis, cryptosporidiosis) are usually mentioned as causative agents of rheumatologic disorders mainly due to immune-mediated mechanisms like reactive arthritis. However, sometimes symptoms are directly related to their infiltration of musculoskeletal structures such as in dracunculiasis, strongyloidiasis (54), filariasis (55), and bilharziasis (56) with a predilection for the ankle and knee. Red flags are endemic areas for parasitosis, poor hygiene conditions, hyper-eosinophilia, immunodeficiencies, and concomitant gastrointestinal or pulmonary involvement. It is important to bear in mind the hurdle of isolating and culture parasites, which require a rare medium, such as Harada-Mori moisture for strongyloidiasis or monkey kidney–mosquito cell lines for filariasis (57).

Whipple disease is another challenge that deserves to be mentioned. In 75% of cases, gastroenteric symptoms are preceded by a seronegative oligo- or polyarthritis with a relapsing course (58), and when the diagnosis remains unclear, biopsy specimen clarify the suspicion showing PAS-positive macrophages beneath the synovial lining cells (59).

Arthritis represents one of the well-known late-stage complications of Lyme disease, especially in the United States. After having investigated prior tick exposure, hazard occupation or hobbies (forestry workers, hunters, and hikers), or previous cutaneous manifestation, the clinician will combine serology and PCR-based testing for B. burgdorferi DNA in the synovial fluid to confirm the diagnosis (60). However, some patients, even after being treated, will develop postinfectious antibiotic-refractory arthritis, where synovial biopsy, which usually shows synovial cell hypertrophy, mononuclear infiltration, vascular proliferation, and sometimes obliterative microvascular lesions, could have a role in the understanding of the chronicity of the process which resembles inflammatory arthritis (61).

Keeping in mind the plethora of the aforementioned microorganisms and their relative hints, once the clinical suspicion arises, the dialogue opens up with the infectiologists, microbiologists, and pathologists to manage properly the analysis of the synovial tissue with their respective culture medium and expected histologic findings (Table 2).

Amidst deposition diseases, there are few which may manifest as monoarthritis, occasionally resembling aggressive osteoarthritis. Ochronosis is an autosomal recessive disorder where the homogentisic acid oxidation products are in excess and therefore deposit in the connective tissue, making it stiffened and brittle, ultimately leading to ochronotic arthropathy. Suspicion should arise if a patient in its fourth decade of life starts having back pain and subsequently knee pain (or hip, shoulder) with radiological findings showing knee osteoarthritis and wafer-like disc calcification with a reduction of intervertebral spaces in the spine. The clinical examination may reveal deposits of bluish or brownish pigment in the ear cartilage and sclerae (62).

Hemochromatosis arthropathy, where iron in excess deposits in the synovial tissue, may virtually involve any joint. The most reported symptoms resemble osteoarthritis and less often recurrent synovitis. Clinical clues comprise the involvement of the second and third metacarpophalangeal joints with their corresponding radiographic findings (hook-like osteophytes), chondrocalcinosis, abnormal liver enzymes, and hyperferritinemia (63).

In the context of monoarthritis, histology remains the gold standard to characterize the nature of proliferative lesions (Table 1). However, most of the time imaging is sufficient to show abnormalities that raise the suspicion and frequently differentiate a local proliferative lesion (50). The topic will be not covered by this review due to its only partial rheumatologic relevance.

After evaluating challenging disease, it is worth mentioning crystal arthropathies, which are supposedly a straight diagnosis. When clinical, instrumental, and synovial fluid analyses are not conclusive, and some uncertainty remains, it must be kept in mind that to indentify crystals under polarizing microscopy, the synovial tissue needs to be processed with absolute alcohol, which is not usually done in the routine analysis, because other fixatives dissolve monosodium urate crystals (45).

Synovial biopsy may be also useful in rheumatic polyarticular disorders (64–68). Osteoarticular manifestations of amyloidosis depend upon the mispleated protein (46). Amyloid light chain (AL) amyloidosis usually presents with an RA-like pattern half of the time associated with cutaneous nodules periarticular or on the extensor surfaces. Bilateral carpal tunnel syndrome is also a frequent finding. Male predominance, the pseudotumoral aspect of the swollen joints, poor response to steroids, radiological evidence of well-circumscribed lytic lesions together with monoclonal gammopathy, macroglossia, hepatomegaly, and peripheral neuropathy should raise the index of suspicion toward amyloidosis. Transthyretin amyloidosis, whether hereditary or senile, mainly manifests as carpal tunnel syndrome due to peripheral neuropathy often starting with sensitive and autonomic symptoms. Other red flags are concomitant arrhythmias and heart failure symptoms.

Likewise, multicentric reticulohistiocytosis mimics RA, progressing up to a mutilans phenotype. It mainly affects women of Caucasian origin in their fourth decade. While laboratory findings are hardly helpful or specific, except by excluding other etiologies, few clinical hints are the involvement of the distal interphalangeal joints, the appearance of papulonodular skin lesions especially affecting the face and hands, and in 25% of cases the association with neoplasia (69).

Finally yet importantly, among the infectious agents, leprosy is one of the diseases where biopsy remains of key importance to demonstrate the presence of the bacilli in the joint. Arthritis in leprosy may be polymorphic, including acute or chronic polyarthritis, septic arthritis, and Charcot's arthropathy. Clinical clues comprise skin lesions and symptoms suggestive of motor-sensory neuropathy in the context of an endemic area (70).

Sarcoidosis is well-known for being polymorphic, with myriads of different musculoskeletal manifestation: acute arthritis (Lofgren syndrome with symmetric hilar adenopathy and erythema nodosum), chronic symmetric, medium to large joint oligoarthritis (especially manifesting with tenosynovitis and skin involvement), up to Jaccoud arthropathy and dactylitis. During the diagnostic management, X-rays could show bone involvement, equally various with different patterns of bone lesions (“moth-eaten,” lytic, and sclerotic lesions). The diagnosis is always challenging, and synovia is one of the precious target tissues that can contribute to it (71).

## Where Should Synovial Biopsy Be Performed?

Synovial biopsy is an invasive procedure; thus, the results expected have to be relevant and informative. All joints are not the same and vary greatly in their vulnerability to different rheumatic diseases. Thus, the choice of the joint to biopsy is crucial and should be guided based on the rheumatologist's purposes.

For example, synovial tissue analysis might be instrumental for the differential diagnosis between rheumatic and non-rheumatic conditions (see paragraph above) of mono-arthritis. In this context, the affected joint should be chosen.

By contrast, in patients with RA, the choice of the joint where synovial biopsy is performed might be based on the published literature (72).

Concerning the inter-joint heterogeneity, in the same patient, it has been demonstrated that synovial samples, taken at the same time, from an active joint are generally representative of other inflamed joints (72, 73). In particular, they provide evidence that cell infiltration of the synovial sublining (i.e., macrophages, T cells, B cells, plasma cells, and IL-6 expression) is similar in large and small joints (74).

In the same patient with RA, a comparison between synovial biopsies in clinically involved and non-involved knee showed that a considerable degree of histological changes, mainly hyperplasia of the synovial lining layer, was present in the uninvolved joint, although changes were less severe than those observed in active joints (72, 73).

Of note, intra-joint variability has also been documented as inflammatory mediators might be differently expressed in different locations of the same joint. In particular, tissue samples from sites close to the cartilage-pannus junction showed a higher level of inflammatory biomarkers that might be underestimated by analyzing specimens from other joint sites (75–77). Although the numbers of T and plasma cells are reported to be similar in biopsy samples (78), one study did find a difference for macrophages (73), but other studies did not confirm this finding (78).

Thus, it is still a matter of debate on the best location from which to obtain synovial tissue samples within a given joint. Recently, an international expert consensus stated that a minimum of four synovial tissue specimens from small joints and six from large joints have to be retrieved for reproducible research studies (79–81), while a previous study showed that using US-guided biopsy of hand joints 12 different samples are recommended to have a valid immunohistochemical assessment (82). During the disease course, the immunohistological features vary when consecutive synovial biopsies from the same joint are analyzed. In the 80s, it has been shown that synovial biopsy of the affected knee in RA patients changes in terms of T and B cells infiltrates according to disease activity when pre- and post-treatment were assessed (39, 51). For this reason, synovial biopsy has also been proposed as a biomarker to evaluate drug response (19, 21, 52).

## How to Perform a Synovial Biopsy

Synovial tissue samples can be retrieved by using different techniques (Table 4) (1). Tissue samples are commonly collected using blind-needle, ultrasound-guided, or arthroscopic-assisted biopsy procedures, but in specific cases, larger synovial samples can also be obtained during an open surgical procedure. In particular, ultrasound-guided needle biopsy (from small and large joints), ultrasound-guided portal and forceps procedures, and arthroscopy are equally successful in sampling synovial tissue and they yield sufficient tissue quantity for transcriptomic studies (83). Moreover, these techniques do not differ in safety or patient tolerability (84). Needle techniques are less invasive for the patients and permit obtaining good-quality synovial tissue in most cases.

**Table 4:** Characteristics of the different techniques used for obtaining a synovial sampling from different joints.

	**Standard arthroscopy**	**Needle arthroscopy**	**Ultrasound needle biopsy**	**Blind needle biopsy**
**Technical issues**				
Synovial sampling success rates	+++	++	++	+
Technically simple	+	++	++	+++
**General issues**				
Acceptability	++	+++	+++	+++
Serial biopsies	+++	+++	+	+
Costs	+++	+++	++	+
**Target joints**				
Large Joints	+++	+++	+++	+++
Small Joints	++	+++	+++	+

### Blind-Needle Biopsy

Blind-needle methods were described in 1950 by Polley et al. with the use of 5-mm-large needles, resulting in a practical but invasive procedure for the modern standards considering that the needle size is similar to the new arthroscopy instrument size. In the years, new and thinner needles have been introduced in the market, simplifying the histologic investigation of articular rheumatic diseases and allowing to perform these procedures without significant pain for the patients, reducing the risk of post-procedural complications (85). Parker and Pearson were the first clinicians to propose a new technique using a composite of two standard items, a 14-gauge thin-walled needle with matching stylet and a 15-gauge aspirating needle with a hook-like beveled tooth that can catch the tissue (40). This instrumentation has been improved in the years to make it more effective, and many semiautomatic guillotine biopsy needles are available on the market to perform a needle biopsy. After disinfection, the skin, subcutaneous tissue, and joint capsule are anesthetized. Anatomical references for the specific joint can be identified with a marking pen to recognize the entry point correctly. Injection of 10–20 cc of isotonic saline into the joint can help the clinicians obtain some material if there is no clinical evidence of effusion. In standard technique, the larger needle is inserted into the joint without a skin incision, and the smaller needle is slipped snugly through it. The needle tip is then entered into the synovial tissue, and its specific hook-design allows to obtain selected tissue when it is withdrawn from the larger needle. Multiple tissue samples can be obtained by changing the direction of the needle (42). This painless procedure gained significant popularity and is considered the basis of modern synovial biopsy techniques due to its numerous advantages like minimal trauma for joint tissues, the possibility of obtaining several samples in one procedure, or performing serial synovial biopsies from the same joint at different times in an outpatient setting. This technique can be performed quickly and with good results in larger joints as the knee; smaller needles can also obtain samples in smaller joints such as the wrist and the ankle. By contrast, the operator cannot have real-time control of the biopsy site. It has been shown that the blind-needle method is less reliable than ultrasound-guided procedures for sampling synovial tissue from the small and large joints (83).

### Ultrasound-Guided Needle Biopsy

Using a blind-needle biopsy technique, the clinicians achieve the procedure without a direct or indirect view, and it is not always possible to have an adequate tissue sample, especially in joints lacking a significant effusion. Some authors described a technique of synovial biopsy under fluoroscopy visualization with a semiautomatic needle. This technique allows the performance of multisite biopsies such as in the hips, shoulders, elbows, ankles, and wrists but requires a complex setting, exposing the patients to x-ray irradiation. Performing harvesting with the aid of ultrasounds could combine the low morbidity of a needle biopsy and the instrumental support's accuracy without ionizing radiation exposure. In recent years, many authors have described good results of ultrasound-guided needle biopsy (43, 44). The skin disinfection and the local anesthesia can be achieved as described in the blind-needle technique; the transducer has to be covered with sterile gel and sterile sheath. The procedure is similar to the already described technique, with all the passages performed under ultrasound control. Authors that published results of this technique described a high success rate of the procedure, with only rare and minor complications (44). Ultrasound-guided needle biopsy and arthroscopic methods are equally successful when sampling synovial tissue from large joints (83).

### Arthroscopic-Guided Synovial Biopsy

Arthroscopic-assisted synovial biopsy is a surgical technique in which the tissue is harvested under the direct view of the suspected pathological area's region, dramatically reducing the risk of mistakes. The technique is a standard joint arthroscopy, with a second portal required to harvest the material of interest using specific instrumentation. This approach's advantages are obtaining more significant macroscopic evaluation pieces, with better sampling from interest areas. Also, arthroscopic synovial biopsy techniques allow biopsies from sites adjacent to the cartilage (47). This area differs quantitatively and qualitatively from the synovium, and collecting tissue with a standard needle technique can be challenging and, in some cases, impracticable. The arthroscopic-assisted technique limits are related to the fact that it is a proper surgical procedure: the need for at least two skin incisions, a longer “learning curve,” and the requirement of a sterile area and operation theater facilities. These procedures are performed in many hospitals by trained orthopedic surgeons, requiring teamwork among different specialists.

### Needle Arthroscopy

A new impressive field of research is needle arthroscopy, where clinicians can use in local anesthesia small modern devices, which permits an exploration of the joint in an outpatient setting and obtain tissue samples under direct view. This well-tolerated procedure allows good macroscopic evaluation of synovial inflammation and selective sampling of the synovial membrane (76).

Finally, surgeons can obtain samples of synovial tissue during an open surgical procedure as a total joint replacement. This technique permits obtaining a relevant quantity of tissue and can help obtain synovial tissue useful for clinical and histological studies in rheumatic patients.

## Conclusion

Within the past decades, several considerable advances have been made in synovial tissue research. Synovial tissue represents the target tissue of many rheumatic and non-rheumatic diseases, and its analysis is crucial in the assessment of many infective, malignancy, and infiltrative disorders. Retrieving synovial tissue samples of good quality using affordable and safe methods from large and small joints is now a realistic desirable objective. Thus, clinical practice pathobiology might be a key component in the management and treatment decision-making process, even in rheumatology.

## Author Contributions

FI, LC, IS, and RCo writing manuscript. RCa and PR revising manuscript. All authors contributed to the article and approved the submitted version.

## Conflict of Interest

The authors declare that the research was conducted in the absence of any commercial or financial relationships that could be construed as a potential conflict of interest.

## Publisher's Note

All claims expressed in this article are solely those of the authors and do not necessarily represent those of their affiliated organizations, or those of the publisher, the editors and the reviewers. Any product that may be evaluated in this article, or claim that may be made by its manufacturer, is not guaranteed or endorsed by the publisher.
